# The efficacy of different torque profiles for weight compensation of the hand

**DOI:** 10.1017/wtc.2023.23

**Published:** 2024-01-29

**Authors:** Bas J. van der Burgh, Suzanne J. Filius, Giuseppe Radaelli, Jaap Harlaar

**Affiliations:** 1 Department of Biomechanical Engineering, Delft University of Technology, Delft, The Netherlands; 2 Department of Precision and Microsystems Engineering, Delft University of Technology, Delft, The Netherlands; 3 Department of Orthopedics & Sports Medicine, Erasmus Medical Center, Rotterdam, The Netherlands

**Keywords:** biomechanics, design, exoskeletons, intelligent orthotics

## Abstract

Orthotic wrist supports will be beneficial for people with muscular weakness to keep their hand in a neutral rest position and prevent potential wrist contractures. Compensating the weight of the hands is complex since the level of support depends on both wrist and forearm orientations. To explore simplified approaches, two different weight compensation strategies (*constant* and *linear*) were compared to the theoretical ideal *sinusoidal* profile and no compensation in eight healthy subjects using a mechanical wrist support system. All three compensation strategies showed a significant reduction of 47–53% surface electromyography activity in the anti-gravity m. extensor carpi radialis. However, for the higher palmar flexion region, a significant increase of 44–61% in the m. flexor carpi radialis was found for all compensation strategies. No significant differences were observed between the various compensation strategies. Two conclusions can be drawn: (1) a simplified torque profile (e.g., constant or linear) for weight compensation can be considered as equally effective as the theoretically ideal sinusoidal profile and (2) even the theoretically ideal profile provides no perfect support as other factors than weight, such as passive joint impedance, most likely influence the required compensation torque for the wrist joint.

## 1. Introduction

Neuromuscular disorders are seriously impacting people’s lives, for example, Duchenne muscular dystrophy (DMD), a genetic disorder that leads to progressive loss of muscle strength (Mercuri et al., 2019). This results in loss of ambulation in early adolescence and progressive loss of arm function, along with the development of joint contractures. Around the age of 12, the functionality and the range of motion (ROM) of the arm decreases (Jannink et al., 2007; Jung et al., 2012).

Compensating for the weight of the arm will add to regain some functionality by reducing the muscular effort to perform a task. A variety of arm-supporting devices already exists to assist the functionality of the shoulder and elbow (Gandolla et al., 2020). A significant reduction ( 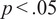 ) in muscle effort of the anti-gravity muscles was shown, such as in the m. biceps brachii ranging from 28 to 60% (Jannink et al., 2007; Iwamuro et al., 2008; Prange et al., 2009; Coscia et al., 2014; Puchinger et al., 2018). However, support of the wrist joint is often neglected, resulting in a flexed rest position of the hand, which may induce wrist contractures. Especially, as the long finger flexor muscles in DMD are prone to shorten (Houwen-Van Opstal et al., 2020). Retaining wrist and finger function is of great importance to perform daily activities and strongly adds to improve quality of life (Houwen-Van Opstal et al., 2020).

To support the weight of the hand, theoretically a sinusoidal torque profile of which the magnitude and phase depend on the hand and forearm orientation is required (see Supplementary Material A). So, a straightforward solution to achieve this would be the use of a counterweight, which directly compensates for the weight of the hand. This yields a considerable (−52%) decrease in extensor muscle activity (Hasegawa et al., 2014). However, the drawback of using a counterweight is that it either uses a large weight or a long lever arm. Such a solution will obstruct movements, look conspicuous, and require additional effort to lift the arm with the added counterweight. A lighter and more slender solution is therefore preferred. Consequently, it is worth considering alternative solutions, that include different torque profiles, that may be equally effective. For example by using linear springs or a constant force. No studies were found that considered weight compensation methods, other than the sinusoidal torque profiles, under different orientations of the forearm.

Therefore, the aim of the current study is to explore whether a constant torque or a linear spring will show similar reductions in muscular effort compared to the theoretically ideal sinusoidal torque profile, considering both the forearm and wrist orientation.

## 2. Method

### 2.1. Participants

For this study, nondisabled participants were recruited from a university student population, with no history of injuries to their right wrist. Ethical approval was obtained from the Human Research Ethics Committee (HREC) at Delft University of Technology (ID2288). All participants gave written informed consent.

### 2.2. Experiment design

**Figure 1.**
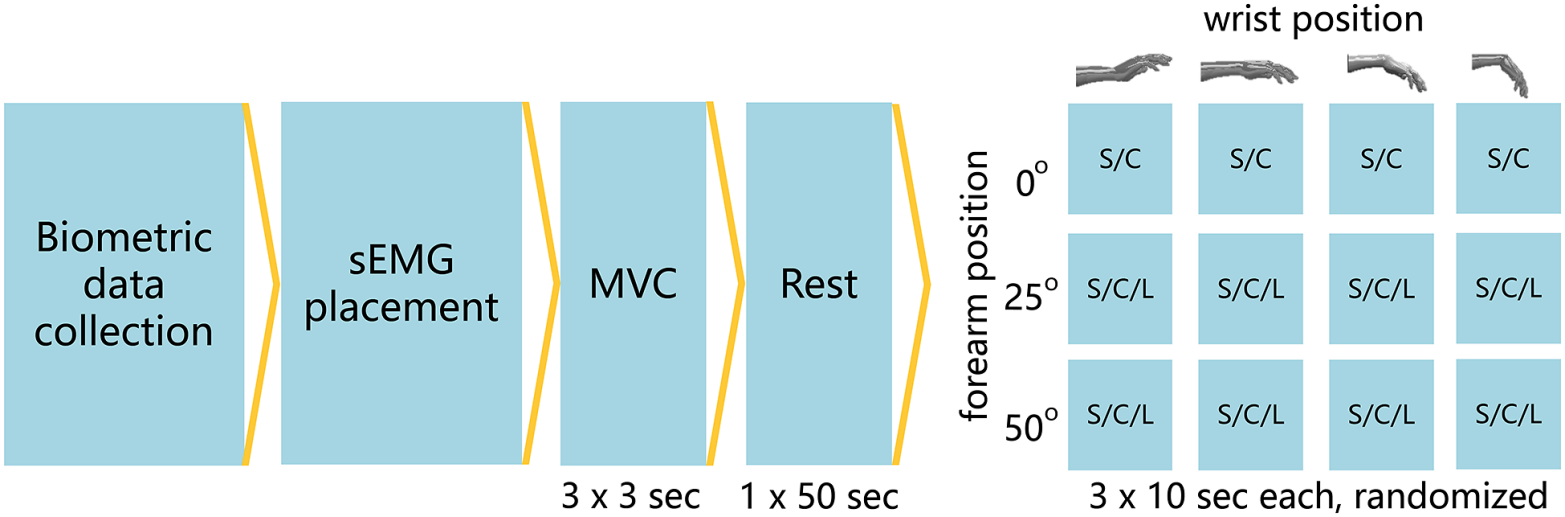
Schematic overview of the experimental design. C, constant; L, linear; MVC, maximally voluntary contraction; S, sinusoidal; sEMG, surface electromyography.

The experiment design is schematically illustrated in Figure 1.

#### 2.2.1. Parameter calibrations

Biometric data collection included the weight and center of mass (CoM) of the hand. The weight of the hand was estimated using a water displacement method (Chandler et al., 1975; Karges et al., 2003) and an anthropometric table (Clauser et al., 1969). The hand CoM was estimated using anthropometric equations including adaptations to adjust for flexed fingers (Clauser et al., 1969; Chandler et al., 1975).

The surface electromyography (sEMG) electrode placement was based on the recommendations from Criswell (2011) and Barbero et al. (2012) for the muscle bellies of the m. extensor carpi radialis (ECR) and the m. flexor carpi radialis (FCR). The reference electrode was placed on the lateral epicondyle of the humerus. The skin preparation was according to the SENIAM recommendations (Hermens et al., 2000), involving shaving and rubbing.

Three maximum voluntary contraction (MVC) tasks were performed by asking the participant to maximally flex or extend their hand against a rigid surface with a straight wrist in a pronated position for 3 s followed by 12 s of rest. During the MVC the participants were encouraged by the experimenter.

A measurement of rest-activity was performed by laying the forearm and hand relaxed on a table in a pronated position for 50 s.

#### 2.2.2. Experiments

For the assessment of the efficacy of the different compensation strategies, the activity of the wrist muscles was evaluated using sEMG for all 12 experimental conditions.

**Figure 2.**
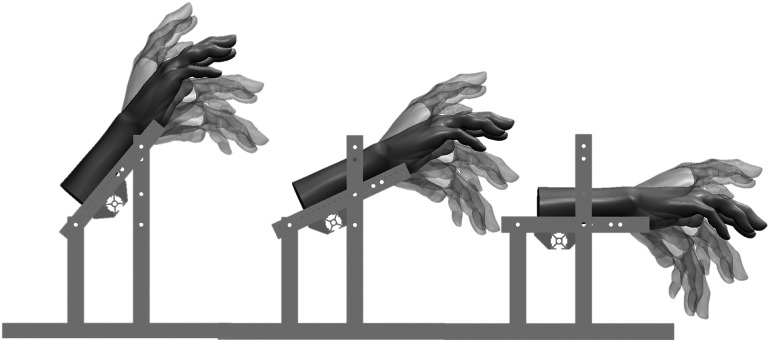
Schematic representation of the different positions of the forearm and hand. From left to right the forearm is positioned at 50, 25, and 0° with respect to the horizontal. The hand can be positioned at 25° dorsal flexion (-25°), neutral (0°), 25° palmar flexion (25°), and 50° palmar flexion (50°) indicated by the transparent hands. Hand model adapted from Story (2020).

The forearm was placed at 0, 25, and 50° with respect to the horizontal. In each successive forearm orientation, the different wrist and balance methods (e.g., constant, linear, sinusoidal, and no support) were evaluated. Within each sEMG recording the wrist was positioned in a pronated position for 10 s at 25° dorsal flexion, 0°, 25°, and 50° palmar flexion (Figure 2), with 5 s rest between each position. Each condition was performed three times in a random order. To control the length of the finger flexors and extensors the position of the fingers was held constant throughout the experiment by gently holding a foam tube during all measurements.

The order of the wrist position and the compensation strategies were randomized to account for the influence of the prior movement direction (Criswell, 2011; Supplementary Material B).

### 2.3. Equipment

#### 2.3.1. Imposing wrist torque profiles

**Figure 3.**
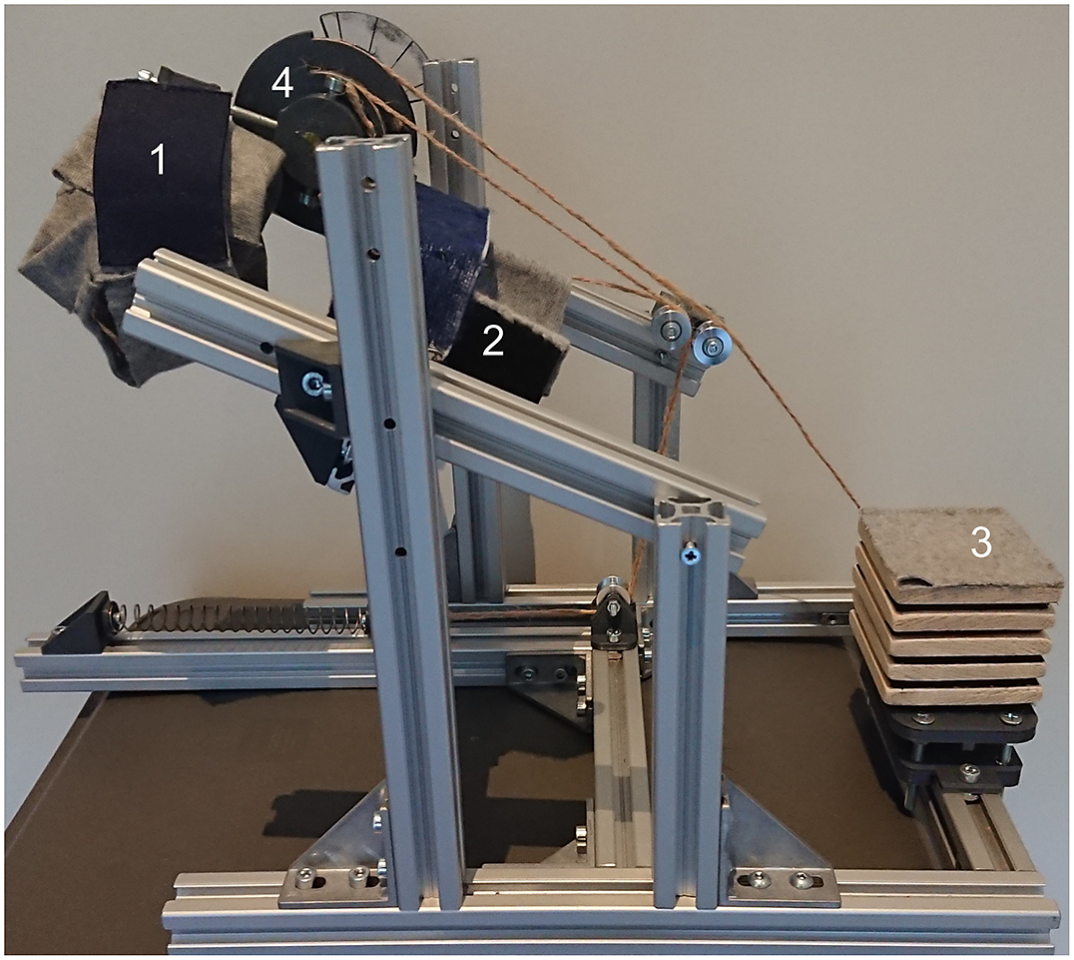
Side view of the setup, (1) hand interface, (2) forearm interface, (3) elbow support, and (4) transmission pulley for application of the different torque profiles.

**Figure 4.**
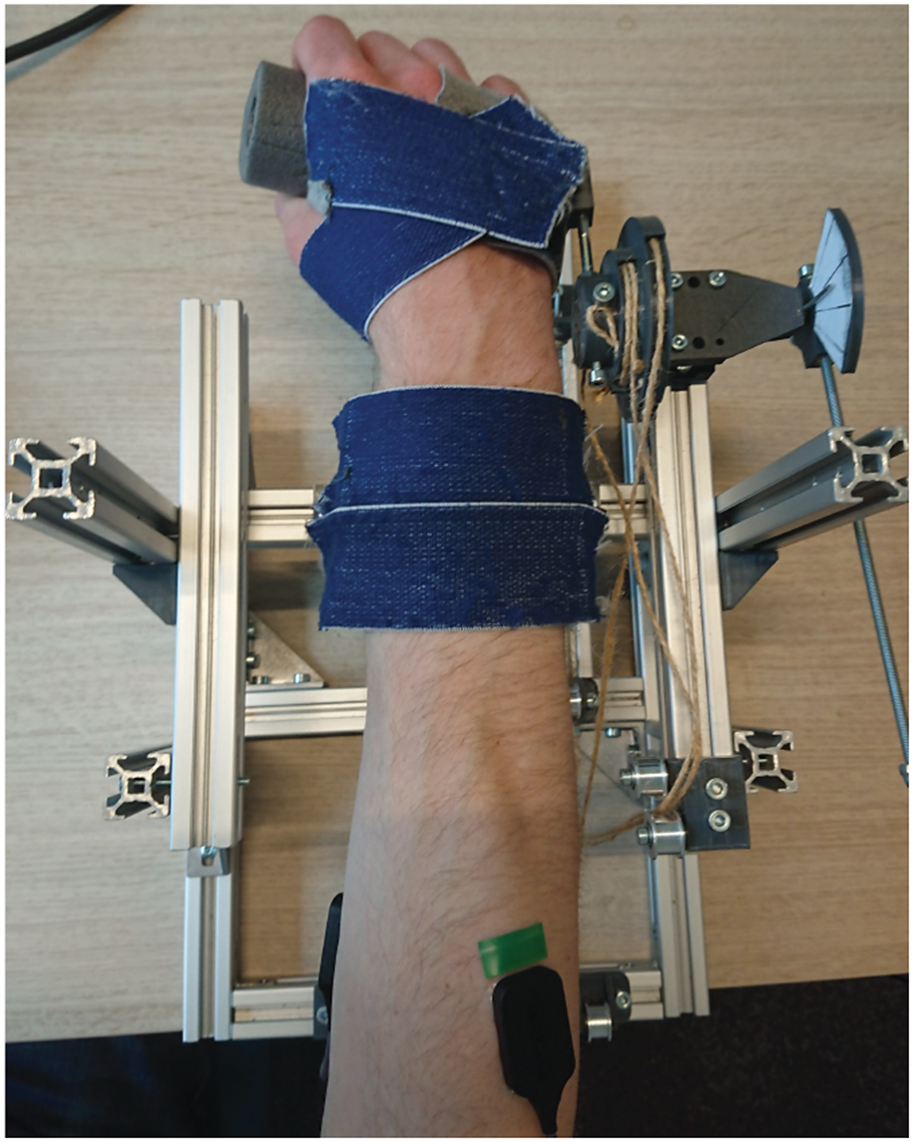
Top view of the setup with arm and sEMG electrodes placed on the ECR and FCR.

**Figure 5.**
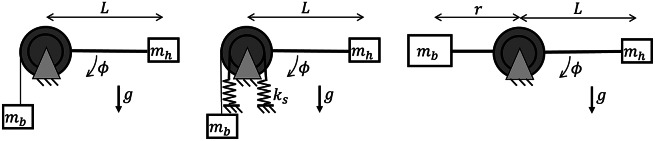
Schematic representation of the application methods of the constant (left), linear (middle), and sinusoidal (right) torque profiles. Here 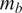 and 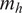 are the balance mass and the mass of the hand respectively and 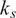 the stiffness of the springs.

**Figure 6.**
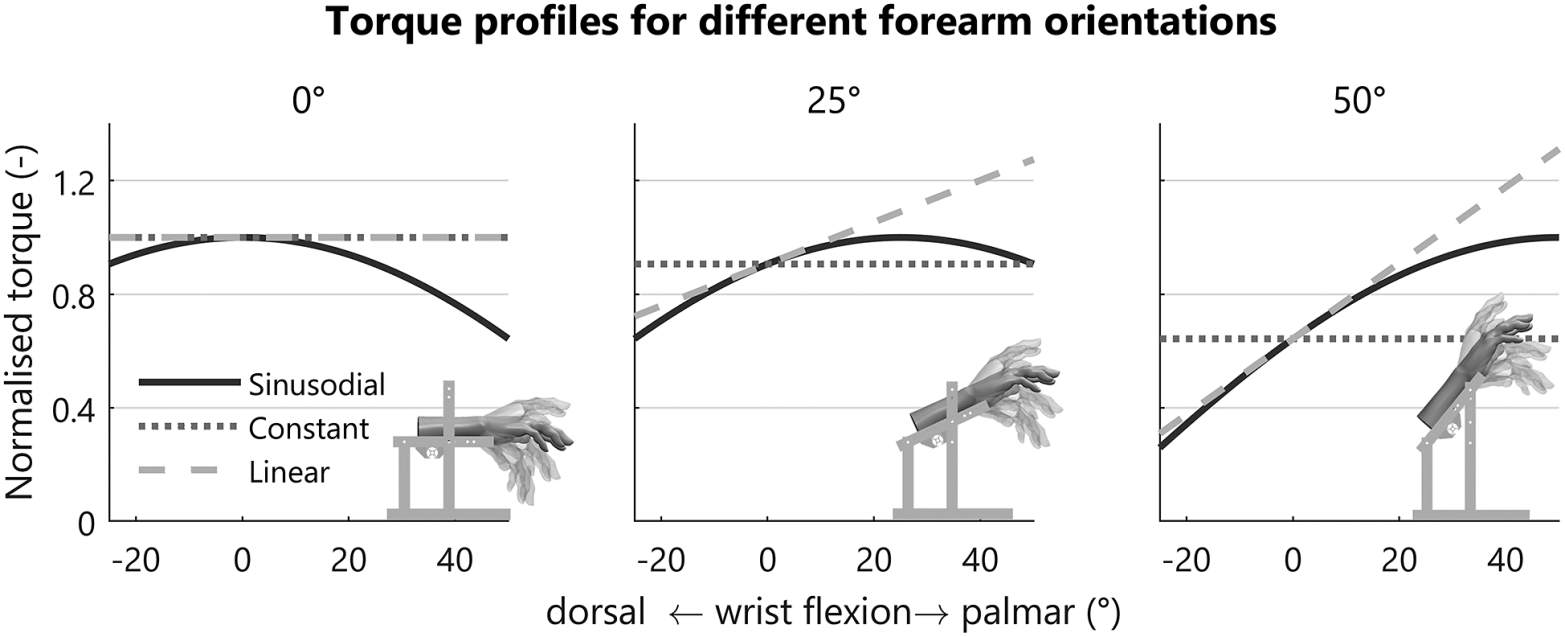
Torque profiles as a ratio of the maximum sinusoidal torque for the three different forearm orientations. The constant torque is only constant with respect to the wrist flexion angle, while it is different for every forearm orientation. The constant and linear torque profiles are respectively the 0th and 1st order expansion of the Taylor series of the sinusoidal profile.

A wrist support system was developed to fixate the forearm in different orientations and provide different compensation torque profiles at the wrist joint (Figures 3 and 4). The human wrist joint was aligned with the system by changing the position of the fixation. The constant torque profile was generated through a mass hanging from a pulley over a constant lever arm (Figure 5). By combining a set of six balance masses (66, 74, 97, 199, 237, 307 g) the desired compensation torque could be approximated within an error of less than 3%. The linear profile was generated by combining the balance masses in series with a spring. By using different types of springs, the slope of the torque profile could be adjusted. For this, a choice was made between four different springs (.15, .19, .25, and .47 N/mm), resulting in an effective stiffness around the wrist joint of .06, .08, .10, or .19 Nm/rad. The linear torque profile was not considered for the forearm orientation of 0° as it behaves as a constant torque. The sinusoidal profile was generated by attaching a counterweight to a rigid rod. By varying the distance of the weight with respect to the hinge of the rod, the magnitude of the profile was adjusted (Figure 5). This value could be adjusted continuously. The required weight compensation torque depends on the biometric properties of the participant’s hand and forearm orientation (Figure 6), as explained in the mathematical wrist model presented in Supplementary Material A.

#### 2.3.2. Surface electromyography

The sEMG recordings were made using a Bagnoli EMG system with DE-2.1 Single Differential Surface EMG sensors from Delsys, consisting of an 8-channel amplifier with an output voltage range of ±5 V (System noise (R.T.I) 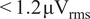 ). The sEMG sensor consists of two 10 mm long and 1 mm wide electrode contacts spaced 10 mm apart (Figure 4). The amplifier gain was set separately for each participant to 1 k or 10 k, maximizing the signal amplitude and avoiding amplifier saturation. The analogue signal was sampled with a frequency of 2000 Hz with a 16-bit (±10 V) ADC NI 9215 (Input noise 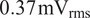 ) using NI LabVIEW 2018. The digitized signal was stored on a computer for offline processing with MATLAB R2021a.

### 2.4. Data processing

The recorded sEMG signal was filtered by applying a second-order IIR Notch filter at 50 Hz to remove power line interference, followed by a sixth order band-pass Butterworth filter with lower and upper cut-off frequencies of 20 and 450 Hz, respectively (Merletti and Cerone, 2020). Then, this signal was rectified by taking its absolute value. Next, the sEMG envelope was defined by taking a third order low-pass Butterworth filter of the rectified signal with a cut-off frequency of 2 Hz (Clancy et al., 2016). Finally, the sEMG envelope of both muscles was normalized with respect to its MVC. The MVC value was defined as the maximum of the sEMG envelope from the three repetitions of the MVC task for both ECR and FCR muscle.

For comparison of the compensation strategies, the mean magnitude of the central 5.5 s of the sEMG envelope for the ECR and FCR muscles was averaged over the three repetitions per condition, expressed in percentage MVC (Burden, 2010; Besomi et al., 2020).

### 2.5. Statistics

For statistical analysis, a repeated measures analysis of variance (ANOVA) is performed on the outcome measures to assess the effects of the forearm orientation, wrist position, and balancing method and their interactions on the sEMG activity of the ECR and FCR muscles. The assumption of sphericity of the data is assessed using Mauchly’s test. If this assumption is violated a Greenhouse-Geisser corrrection was applied to the degrees of freedom to account for this violation. Following the ANOVA, the results are analyzed using a multiple comparison with Bonferroni adjustment (Field, 2013). *p*-values 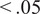 are interpreted as significant. Data are reported as mean ± standard deviation. Statistical analyses were performed using IBM SPSS Statistics for Windows, Version 28.0.1.0 (Armonk, NY, USA: IBM Corp.).

## 3. Results

### 3.1. Participants

Nine people participated in the experiment (see Table 1). One participant had to be excluded due to a technical error during the experiment. All participants were right-handed.

**Table 1.** Participant characteristics are reported as mean and standard deviation

Characteristic	Male (*N* = 4)	Female (*N* = 4)
Age (years)	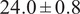	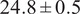
Body weight (kg)	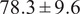	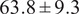
Height (m)	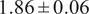	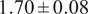
Hand weight (g)	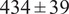	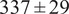
Centre of mass (mm)	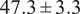	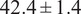

### 3.2. sEMG activity

**Figure 7.**
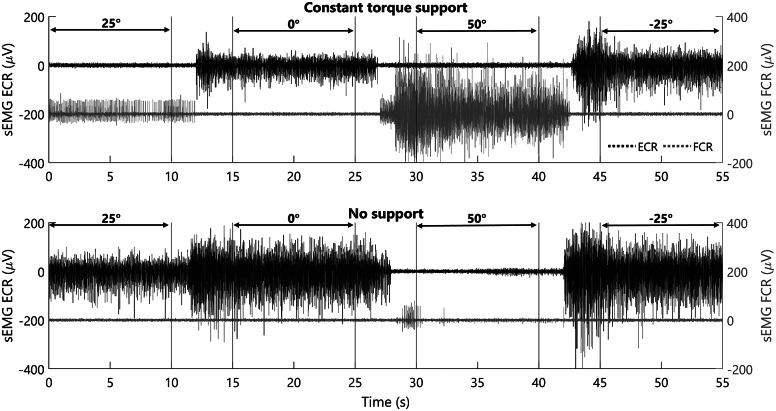
Raw sEMG data from one participant for a single measurement of constant torque compensation (top graph) and no compensation (bottom graph), for different levels of palmar flexion with the forearm at 0°. Note that the order of the wrist position is randomly assigned throughout the experiments. However, for clarity, the results of two experiments of the same participant are depicted which follow the same order.

**Figure 8.**
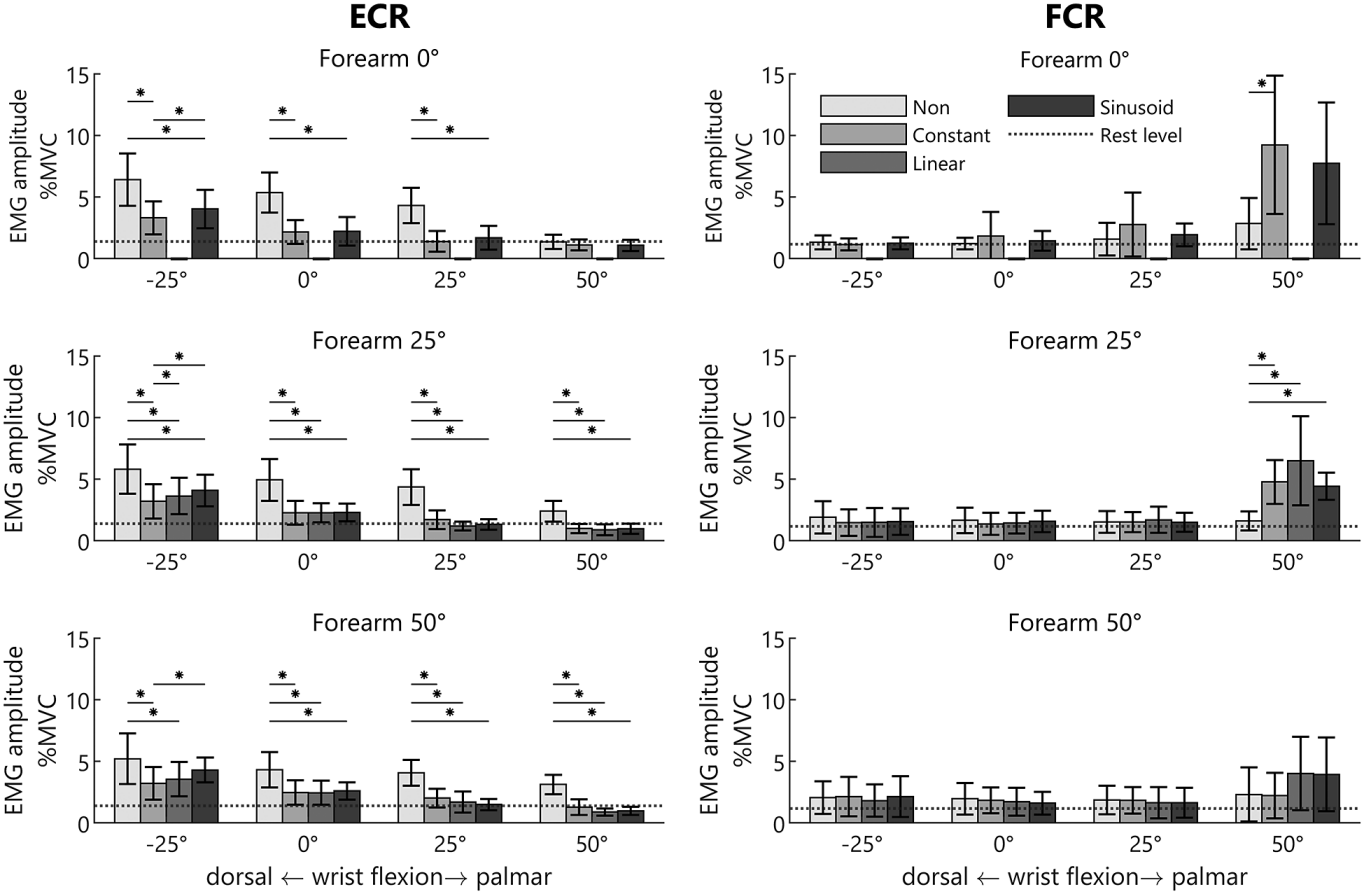
Mean sEMG magnitude relative to the MVC of the ECR (left) and FCR (right) for different forearm positions, wrist positions, and balance methods. Reported as mean and standard deviation. *indicates a statistically significant difference. Note, for the 0° forearm position the linear torque profile is combined with the constant torque profile as they are the same for this orientation (therefore no separate measurements were performed).

A representative example of the raw sEMG data is depicted in Figure 7 for the constant torque and no compensation. Overall, the ECR activity is lower for the constant torque support, while the FCR activity increases for larger levels of palmar flexion. Additionally, from the figure it can be observed that the signal is approximately constant throughout the central 5.5 s segment in each condition, showing its validity to be used in the calculation of the outcome measure. The combined results for the different participants for each condition can be found in Figure 8.

#### 3.2.1. Extensor carpi radialis

Using Mauchly’s test it was observed that the assumption of sphericity had been violated for the main effects of wrist position for the ECR, 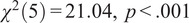 and balance method 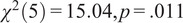 . Consequently, the degrees of freedom were corrected using Greenhouse-Geisser estimates of sphericity. The effect of wrist position and balance method was observed to be significant (*p* < .001). A pairwise comparison showed that the ECR activity on average reduced (*p* < .001) with 51% for constant ( 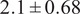 %MVC), 53% for linear ( 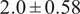 %MVC), and 46% for sinusoidal ( 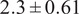 %MVC) compared to no compensation ( 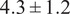 %MVC). This can also be seen from Figure 8 showing an overall decrease in ECR activity close to rest level. No significant effects were observed between the different types of compensation.

#### 3.2.2. Flexor carpi radialis

Using Mauchly’s test it was indicated that the assumption of sphericity had been violated for the main effects of wrist position for the FCR, 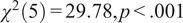 . Consequently, the degrees of freedom were corrected using Greenhouse-Geisser estimates of sphericity. The effect of wrist position (*p* < .001), balance method (*p* < .001), and forearm orientation (*p* = .008) were observed to be significant. A pairwise comparison showed that the FCR activity on average increased with 50% (*p* = .012) for constant ( 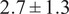 %MVC), 61% (*p* = .005) for linear ( 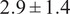 %MVC), and 44% (*p* = .005) for sinusoidal ( 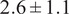 %MVC). This is mainly caused by a large increase in FCR activity at 50° palmar flexion when a form of compensation is used, especially at the horizontal forearm position, see Figure 8. Considering all forearm orientations, the FCR activity increased with 134% (from 2.3 to 5.4%MVC) for the constant and sinusoidal torque profile and 187% (from 2.3 to 6.6%MVC) for the linear torque profile with respect to no compensation when flexing the wrist from 0° to 50°. No significant effects were observed between the different types of compensation.

## 4. Discussion

This study was conducted to assess whether two simplified weight compensation strategies show a similar muscle activity reduction as the theoretically ideal sinusoidal strategy applied in a wrist support system to compensate for the weight of the hand. Based on the pairwise comparison between the different compensation strategies combining all wrist and forearm orientations, for both the FCR and ECR, a significant difference was found between with and without compensation, while no differences were found between the different compensation strategies (constant, linear and sinusoidal). These findings indicate that the simplified constant and linear torque profiles can be regarded effective alternatives for compensation, as opposed to the sinusoidal torque profile. These findings could not be compared with literature as to the authors’ knowledge there are no studies that systematically investigate the influence of different types of compensation on the activity of the wrist muscles.

Although the anti-gravity ECR muscle showed a significant reduction in muscle activity in almost all configurations, the FCR showed only an increase at 50° palmar flexion within all compensation strategies. Although this increased activity is relatively small ( 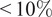 MVC), it indicates that there is an overcompensation of the required compensation at 50° palmar flexion. For the constant and linear compensation strategies, this overcompensation could be explained by overestimation of the required compensation torque resulting from the simplification of the theoretical required torque, see Table 2. However, in case of the sinusoidal profile (and the constant profile at the 25° forearm orientation, i.e., C25) no overestimation of the theoretical required compensation is provided and still an increase of the FCR activity was detected. This suggests that for the higher levels of palmar flexion, a lower theoretical compensation torque is required than what follows from the theoretically ideal sinusoidal strategy. This mismatch between the actual and theoretical compensation torque could be explained by the effects of passive joint impedance. Each joint in the human body demonstrates resistance against passive motion, especially near the end range of motion, resulting from muscles, ligaments, nerves, blood vessels, and skin (Chleboun et al., 1997; Ayhan and Ayhan, 2020). Formica et al. (2012) measured the passive joint impedance of the wrist joint in healthy subjects. From their results it can be observed that for larger palmar flexion angles the magnitude of the passive joint impedance becomes equal to or even larger than the gravitational moments. Consequently, it is hypothesized that the increase in FCR activity for the higher flexion region is (partially) a result of this inherent passive joint impedance. This assumes that when no support is provided the moments generated by gravity are large enough to overcome at least partially the passive joint impedance during flexion. So, when the weight of the hand is compensated, additional forces are required to palmar flex the hand to overcome the passive joint impedance to reach a palmar flexion angle of 50°, resulting in elevated FCR activity. The influence of this joint impedance is especially of importance when translating these findings to people which suffer from an increased passive joint impedance which is often the case in chronic stroke, spinal muscular atrophy, and MD patients (Schmit et al., 2000; Cornu et al., 2001; Ragonesi et al., 2013; de Gooijer-van de Groep et al., 2016).

**Table 2.** Level of compensation of the simplified balancing methods compared to the theoretical required torque (e.g., sinusoidal profile) for the specific wrist and forearm positions

	Wrist position			
Balance method	−25 (%)	0 (%)	25 (%)	50 (%)
C 0	110	100	110	156
L 0	110	100	110	156
C 25	141	100	91	100
L 25	112	100	109	141
C 50	248	100	71	64
L 50	119	100	108	131

*Note.* Larger values than 100% indicate overcompensation whereas smaller values indicate under compensation. For the balance method, C = constant and L = linear, while the adjoining number indicates the forearm orientation. A more general overview of the level of compensation is depicted in Figure 6.

### 4.1. Limitations of the study

When inspecting the data presented in Figure 8, it can be observed that certain conditions exhibit a substantial standard deviation. This is especially the case for the FCR. This is likely caused by the fact that the activity was close to rest level, making it more susceptible to noise (e.g., power line interference). Additionally, some variation was potentially caused by estimation errors of the hand weight and CoM (see Supplementary Material C; Coscia et al., 2014; Runnalls et al., 2014). However, the estimated masses are comparable to literature (Clauser et al., 1969; Durkin and Dowling, 2003). Moreover, the resolution of the applied torques for the constant and linear profiles was finite. This was especially a limitation for the linear profile, due to the limited number of springs available. For some participants, this finite resolution resulted in a small deviation from the required compensation estimated. When considering the results of all participants across all experiments, on average the differences between the applied and desired compensation were 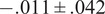 N/mm for the stiffness and 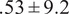 g for the weight, resulting in a difference of less than 10%. Other possible sources for spread in the results might be due to variation in the passive joint impedance and potential misalignment of the human wrist joint with respect to the mechanical joint of the set-up.

The human wrist deviates from that of an ideal hinge joint (Ayhan and Ayhan, 2020). While the impact of this deviation is minimal at smaller flexion angles, it becomes more prominent as the angles increase, due to translation of the axis of rotation of the human wrist joint (Schiele and van der Helm, 2006). Joint misalignment can introduce interaction forces between the human and the set-up, such as shear forces at the interface, requiring more effort to flex the wrist. Moreover, correctly palpating the muscle belly of the FCR was challenging in some participants, potentially resulting in a less ideal sensor placement, yielding cross-talk from other muscles.

### 4.2. Recommendations

This study determined the constant and linear profile by making use of a Taylor expansion around the neutral position of the wrist (0° flexion) of the sinusoidal profile (Supplementary Material A). However, other approximations, for instance taking an average of the profile, are possible directions for additional investigation for further optimization of the provided level of support. However, more importantly, the influence of passive joint impedance should be taken into account, since the passive joint impedance plays a substantial role in the required level of compensation, especially in many clinical cases and near the joint limits.

## 5. Conclusion

This study has shown that simplified torque-angle profiles (e.g., constant or linear) can be considered as an alternative to the conventional sinusoidal profile to compensate for the weight of the hand. However, the increase in FCR activity for all compensation methods, including the conventional sinusoidal, indicates a limitation of the applied compensation strategies as they do not account for passive joint impedance near the joint limits. As such, more research is needed to assess this influence of passive joint impedance on the required compensation profile. Meanwhile, the findings of this study can be used to inform the development of simplified wrist supports for people suffering from (neuro)muscular weakness.

## Supporting information

Van Der Burgh et al. supplementary material Van Der Burgh et al. supplementary material

## Data Availability

The data and materials allowing users to understand, verify, and replicate the findings are published under restricted access on the 4TU. ResearchData repository: http://doi.org/10.4121/71912bfe-81ad-4e9f-ad92-30a430594f69, and can be accessed upon request.

## Supplementary material

The supplementary material for this article can be found at https://doi.org/10.1017/wtc.2023.23.
