# Supplementary material for: The efficacy of different torque profiles for weight compensation of the hand
Source: Wearable Technol. 2024 Jan 29;5:e2. doi: 10.1017/wtc.2023.23 (PMC10952050; doi:10.1017/wtc.2023.23)
Supplement: Van Der Burgh et al. supplementary material [file S2631717623000233sup001.pdf]

Supplementary material: The efficacy of different torque profiles for weight compensation of the hand

B.J. van der Burgh[1,2], S.J. Filius[1*], G. Radaelli[2] and J. Harlaar[1,3]

[1]Department of Biomechanical Engineering, Delft University of Technology, Delft, The Netherlands. [2]Department of Precision and Micro Engineering, Delft University of Technology, Delft, The Netherlands. [3]Department of Orthopedics & Sports Medicine, Erasmus Medical Center, Rotterdam, The Netherlands.
*Corresponding author. E-mail: s.j.filius@tudelft.nl

Supplementary material A: Torque models

When looking at current wrist supports [1, 2], which aim to support the hand against gravity, such as the Ambroise Dynamic Wrist Orthosis [3], they primarily focus on supporting palmar-dorsal flexion of the wrist. As such gravity compensation of the hand for palmar-dorsal flexion is considered here. Consequently, as only one degree of freedom of the wrist is considered it can be simplified as a revolute joint. Thus, the required torque to compensate the weight of the hand can be expressed as

$$T = mgL\cos(\theta - \phi)\cos\psi \tag{1}$$

Where m is the mass of the hand, L the distance from the wrist joint to the hand's centre of mass, g the gravitational acceleration, ϕ the angle of the wrist with respect to the forearm, θ the inclination of the forearm and ψ the pronation-supination angle at zero inclination (Fig. 1). The weight of the hand can be perfectly compensated by attaching a balance weight opposite to the centre of mass (Fig. 2), resulting in a sinusoidal torque profile. To simplify the required torque profile, it can be approximated using Taylor expansions. Here the 0th and 1st order expansion will be considered. The 0th order expansion around the neutral wrist position $\phi_0 = 0$ can be expressed as

$$T = mgL\cos\theta\cos\psi \tag{2}$$

From this it can be noticed that the required torque only depends on the orientation of the forearm and no longer on the position of the wrist. Thus, the torques are constant for each position of the wrist. The 1st order expansion is given by

$$T = mgL(\cos\theta + \phi\sin\theta)\cos\psi \tag{3}$$

Consequently, the torque depends linearly on the wrist position. This linear term can be considered as a torque generated by a linear spring with torsional stiffness $k_\phi = mgL\sin\theta$ or $k = mg\frac{L}{r^2}\sin\theta$ for an ordinary coil spring attached to a pulley of radius r.

The orientations considered during the experiment correspond to ψ=0°, θ=0, 25, 50° and ϕ=-25, 0, 25, 50°. The angle ψ is achieved physically through a combination of predominantly pronation of the forearm and a slight abduction of the shoulder. The different angles of θ are achieved through a combination of elbow and shoulder flexion. The different angles of ϕ are achieved through dorsal and palmar flexion of the wrist.

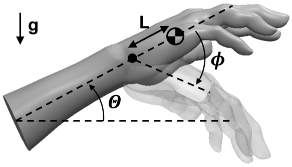

Figure 1. Model of the hand. Note that ϕ is chosen positive for palmar flexion. Hand model adapted from [4].

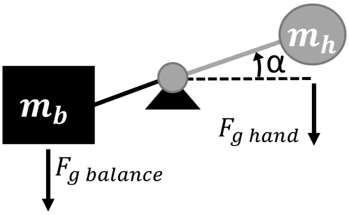

Figure 2. Example of a gravity balancer using a balance weight, which generates a sinusoidal torque profile as a function of α around the joint.

Supplementary material B: Effect of movement direction

To assess the effect of the movement order of the wrist on the muscle activity of the m. extensor carpi radialis and the m. flexor carpi radialis a separate experiment was performed. This experiment was performed with one subject and each measurement was performed 10 times. The participant was asked to move his hand form 25° dorsiflexion to 0° and then to 25° and 50 ° palmar flexion, after following the same order backwards. The orientation of the forearm was held constant throughout the experiment and no support was provided.

The biometric data of the participant are depicted in Table 1. The results of the experiment are depicted in Fig. 3 showing in general a higher activity when moving from palmar to dorsal (against gravity) than from dorsal to palmar (with gravity). The first case (dorsal to palmar flexion) involves a concentric contraction (muscle shortens during contraction) while the second (palmar to dorsal flexion) involves an eccentric contraction (muscle lengthens during contraction). These results are also expected as in general the measured activity during a concentric contraction is larger than during eccentric contraction [5].

Table 1. Participant characteristics, reported as mean and standard deviation.

Gender	Age (years)	Weight (kg)	Height (m)	Hand weight (g)	Centre of mass (mm)
Male	24	61	1.88	353	43.6

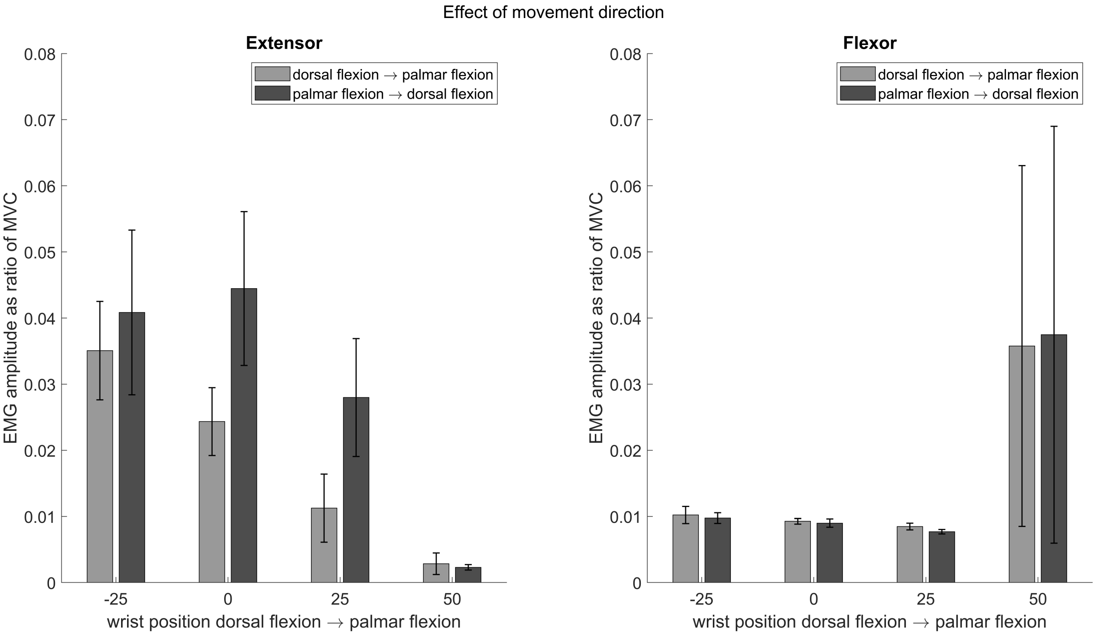

Figure 3. Mean normalised EMG-amplitude of the m. extensor carpi radialis (left) and m. flexor carpi radialis (right) for two movement directions. Error bars are one standard deviation.

Supplementary material C: Effect of different levels of compensation

To assess the effect of different levels of compensation an additional experiment was performed for the constant torque by using different weights. This experiment was performed with one subject and each measurement was performed five times. In sets of three and two. The order of the level of compensation was randomised as well as the order of the wrist positions. The orientation of the forearm was held constant throughout the experiment.

The biometric data of the participant are depicted in Table 1. The results of the experiment are shown in Fig. 4. From this it can be concluded that using more compensation results in a larger reduction in the activity of the anti-gravity muscle (extensor). However, a larger level of compensation results in an increase in the flexor muscle activity for palmar flexion. This can also be expected as for larger levels of compensation overcompensation occurs, requiring additional effort of the flexor muscles. When comparing these findings with literature, Coscia et al. observed a decrease in muscle activity of the anti-gravity muscles when increasing the level of support from no to full support [6]. Here they used perfect balancing which is comparable to the sinusoidal profile as discussed in this article. Note that in this experiment non-perfect balancing is used (a constant torque) and the levels are increased beyond the full compensation. A similar effect was observed by Runnals et al. [7]. Comparing the results of this additional experiment with the main experiment it can be observed that for the extensor the outcomes are similar when looking at 0% and 98% support. However, the flexor amplitude is considerably higher when support is used, compared to the mean results from the main experiment. However, this is likely caused by interindividual differences as also in the main experiment some participants showed similar results.

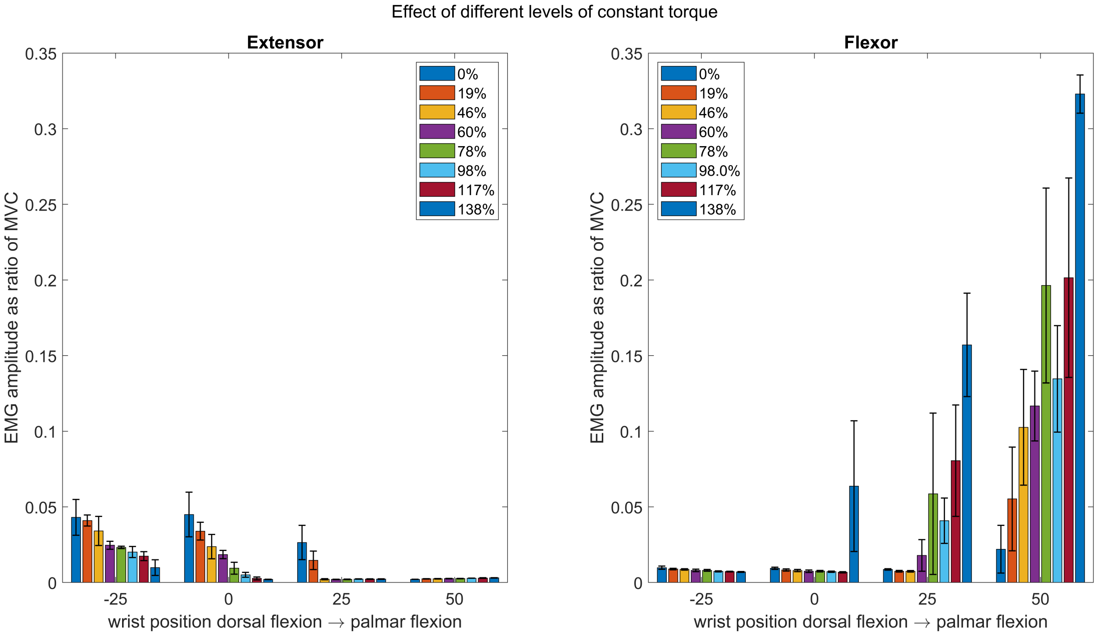

Figure 4. Mean normalised EMG amplitude for different levels of constant torque compensation of the extensor carpi radialis (left) and flexor carpi radialis (right). Error bars are one standard deviation.

References

1. Heo, P., Gu, G. M., Lee, S.-j., Rhee, K. & Kim, J. Current hand exoskeleton technologies for rehabilitation and assistive engineering. *International Journal of Precision Engineering and Manufacturing* **13,** 807–824. ISSN: 2234-7593. http://link.springer.com/10.1007/s12541-012-0107-2 (May 2012).
2. Bos, R. A. *et al.* A structured overview of trends and technologies used in dynamic hand orthoses. *Journal of NeuroEngineering and Rehabilitation* **13,** 62. ISSN: 1743-0003. http://jneuroengrehab.biomedcentral.com/articles/10.1186/s12984-016-0168-z (Dec. 2016).
3. *Ambroise Dynamische Polsorthese • Ambroise* https://www.ambroise.nl/armorthesen/polsorthesen/ambroise-dynamische-polsorthese/.
4. Story, A. *Right Hand Reference* 2020. https://grabcad.com/library/right-hand-reference-1.
5. Criswell, E. C. J. R. *Cram's introduction to surface electromyography* English. ISBN: 9780763732745 (Jones and Bartlett, Sudbury, MA, 2011).
6. Coscia, M. *et al.* The effect of arm weight support on upper limb muscle synergies during reaching movements. *Journal of NeuroEngineering and Rehabilitation* **11,** 22. ISSN: 1743-0003. http://jneuroengrehab.biomedcentral.com/articles/10.1186/1743-0003-11-22 (2014).
7. Runnalls, K. D., Anson, G., Wolf, S. L. & Byblow, W. D. Partial weight support differentially affects corticomotor excitability across muscles of the upper limb. *Physiological Reports* **2,** e12183. ISSN: 2051817X. http://doi.wiley.com/10.14814/phy2.12183 (Dec. 2014).
